# The Influence of Hepatitis B Viral Load and Pre-S Deletion Mutations on Post-Operative Recurrence of Hepatocellular Carcinoma and the Tertiary Preventive Effects by Anti-Viral Therapy

**DOI:** 10.1371/journal.pone.0066457

**Published:** 2013-06-21

**Authors:** Chien-Wei Su, Yu-Wei Chiou, Yi-Hsuan Tsai, Ruei-Dun Teng, Gar-Yang Chau, Hao-Jan Lei, Hung-Hsu Hung, Teh-Ia Huo, Jaw-Ching Wu

**Affiliations:** 1 Division of Gastroenterology, Department of Medicine, Taipei Veterans General Hospital, Taipei, Taiwan; 2 Faculty of Medicine, Cancer Research Center, National Yang-Ming University, Taipei, Taiwan; 3 Institute of Clinical Medicine, Cancer Research Center, National Yang-Ming University, Taipei, Taiwan; 4 Department of Medical Research and Education, Taipei Veterans General Hospital, Taipei, Taiwan; 5 Division of General Surgery, Department of Surgery, Taipei Veterans General Hospital, Taipei, Taiwan; 6 Division of Gastroenterology, Department of Medicine, Cheng Hsin General Hospital, Taipei, Taiwan; 7 Institute of Pharmacology, National Yang-Ming University, Taipei, Taiwan; Johnson & Johnson Medical, China

## Abstract

**Background:**

Whether or not hepatitis B virus (HBV) genotypes, mutations, and viral loads determine outcomes for patients with HBV-induced hepatocellular carcinoma (HCC) remains controversial.

**Aims:**

To study the influence of HBV viral factors on prognoses for patients with HBV-induced HCC after resection surgery and investigate if antiviral therapy could counteract the adverse effects of viral factors.

**Methods:**

A total of 333 HBV-related HCC patients who underwent tumor resection were enrolled retrospectively. Serum HBV DNA levels, mutations, anti-viral therapy, and other clinical variables were analyzed for their association with post-operative recurrence.

**Results:**

After a median follow-up of 45.9 months, 208 patients had HCC recurrence after resection. The 5-year overall survival and recurrence-free survival rates were 55.4% and 35.3%, respectively. Multivariate analysis showed indocyanine green retention rate at 15 minutes >10%, gamma-glutamyltransferase (GGT) level >60 U/L, macroscopic and microscopic venous invasion, and the absence of anti-viral therapy were significant risk factors for recurrence. Anti-viral therapy could decrease recurrence in patients with early stage HCC, but the effect was less apparent in those with the Barcelona-Clinic Liver Cancer stage C HCC. For patients without antiviral therapy after resection, serum HBV DNA levels >10^6^ copies/mL, GGT >60 U/L, and macroscopic and microscopic venous invasion were significant risk factors predicting recurrence. Among the 216 patients without anti-viral therapy but with complete HBV surface gene mapping data, 73 were with pre-S deletion mutants. Among patients with higher serum HBV DNA levels, those with pre-S deletion had significantly higher rates of recurrence. Moreover, multivariate analysis showed multi-nodularity, macroscopic venous invasion, cirrhosis, advanced tumor cell differentiation, and pre-S deletion were significant risk factors predictive of recurrence.

**Conclusions:**

Ongoing HBV viral replication and pre-S deletion are crucial for determining post-operative tumor recurrence. Anti-viral therapy can help reduce recurrence and improve prognosis, especially for those with early stage HCC.

## Introduction

Hepatocellular carcinoma (HCC) is the third leading cause of cancer mortality worldwide [1]. Surgical resection is the major treatment modality for patients with early-stage HCC and well-preserved liver function [1]–[3]. However, the long-term outcome after resection remains unsatisfactory because of the high post-operative recurrence rate, which accounts for the high mortality [4], [5]. To improve prognosis, risk factors affecting hepatocarcinogenesis and post-surgical recurrence of HCC must be identified.

Tumor size and number, presence of venous invasion, and liver functional reserve are reported to determine post-operative tumor recurrence [2], [6], [7]. As regards viral factors, hepatitis B virus (HBV) is the most common etiological factor of HCC, especially in Asia [1], [8]. The pathogenesis of HCC in chronic HBV infections has been investigated comprehensively in recent years. The REVEAL-HBV cohort study demonstrates a significant dose-response relationship between serum HBV DNA levels and the risk of developing HCC in chronic HBV carriers [9]. In addition to viral load, HBV genotype and basal core promoter (BCP) mutations are also correlated with the development of HCC [10], [11]. However, whether or not HBV genotypes, mutations, and viral loads determine outcomes for patients with HBV-induced HCC after resection surgery remains controversial [2], [12]. Moreover, the efficacy of anti-viral therapy after resection is still debated [13], [14].

There are three forms of hepatitis B surface antigen (HBsAg) composition: large (coded for by the pre-S1/pre-S2/S gene), middle (the preS2/S gene), and small (the S gene) protein [15]. The pre-S region of the HBsAg carries several potent T and B cell epitopes [16]. During the natural history of chronic hepatitis B, viral variants and deletion mutants may be selected by immune response. Cross-sectional studies have demonstrated that patients with HBV-induced HCC had significantly higher frequencies of harboring pre-S deletion mutants than HBV carriers without HCC [17], [18]. Longitudinal cohort studies also show that the presence of pre-S deletion mutants in chronic hepatitis B patients is a significant risk factor of developing HCC during follow-up [19]. While these reports suggest a close relationship between pre-S deletion mutants and hepatocarcinogenesis, the prognostic value of pre-S deletion mutants in HCC patients after curative therapies is still unclear due to the relatively small size of patient number with pre-S deletion mutants in the previous studies [20], [21].

This study aimed to comprehensively elucidate the impact of ongoing HBV viral replication and viral mutations on postoperative HCC recurrence, and the tertiary chemo-preventive effects of anti-viral therapy on recurrence.

## Materials and Methods

### Patients and Follow-up

There were 607 consecutive treatment-naïve HBV-related HCC patients who underwent curative resection surgery in Taipei Veterans General Hospital from 1990 to 2007. The inclusion criteria were (a) positive hepatitis B surface antigen (HBsAg) in sera; (b) liver function of A or B by Child’s classification, with an indocyanine green 15-minute retention rate (ICG-15R) <30%; (c) tumors involving no more than three Healey’s segment without portal vein main trunk involvement; (d) absence of other major diseases that might complicate surgery; (e) absence of extra-hepatic tumor dissemination [6], [22]. Among them, 333 patients who had stored serum samples available for virological analysis were enrolled in this study. The demographic characteristics between patients with and those without virological data were compared in **Table S1**. The demographic characteristics were similar between these two groups except patients who did not have detail virological data had larger tumor size than those with detail HBV virological data. No other forms of adjuvant anti-tumor therapy such as local ablation therapy, chemoembolization, or molecular target therapy, were performed before or after resection until the emergence of tumor recurrence. Patients with concurrent infection of hepatitis C virus (HCV) or hepatitis D virus (HDV) were excluded.

After surgery, the macroscopic features of the tumor, including size, number, and vascular invasion were assessed. Histologically, microscopic vascular invasion and cirrhosis of the non-tumor part were examined by microscopy. Cancer cell differentiation was determined by Edmondson grade [23].

After surgery, patients visited outpatient clinics regularly every three months and assessed by testing serum liver biochemistries and AFP levels, and ultrasonography. Tumor recurrence was suspected if serum alpha-fetoprotein (AFP) levels were elevated (>20 ng/mL) or if new lesions were detected by surveillance ultrasonography. These lesions were further diagnosed by dynamic computed tomography or magnetic resonance imaging with presence of typical vascular pattern for HCC.

The Institutional Review Board of the Taipei Veterans General Hospital has approved the study protocol and consent procedure, which complied with the standards of the Declaration of Helsinki. All of the patients provided their written informed consent to participate in this study.

### Biochemical and Serological Markers

Serum HBsAg levels were quantified by Architect HBsAg QT (Abbott Diagnostic, Germany) according to the manufacturer instructions. Hepatitis B e antigen (HBeAg), antibody against HBeAg (anti-HBe), and antibody to HDV were tested using a radio-immunoassay kit (Abbott Laboratories, North Chicago, IL), while anti-HCV was assessed by a second-generation enzyme immunoassay kit (Abbott Laboratories, North Chicago, IL).

Serum biochemistries were measured using the Roche/Hitachi Modular Analytics Systems (Roche Diagnostics GmbH, Mannheim, Germany) and serum AFP level was tested by radio-immunoassay (Serono Diagnostic SA, Coinsin/VD, Switzerland).

### Detection and Quantification of HBV DNA, HBV Genotyping and Sequencing

The complete HBV envelope open-reading frame was amplified using a pair of primers B2822(+) (5′-ggg,TCA,CCA,TAT,TCT,Tgg-3′) and B840R (5′- ACC,CCA,TCT,TTT,TgT,TTT,gTT,Agg-3′) that covered the entire coding region of pre-S1, pre-S2, and S gene (2848–3215 and 1–835) via polymerase chain reaction (PCR). The PCR was performed in a thermal cycler (Perkin Elmer Cetus Corp., Norwalk, CT), beginning at 95°C for 5 min, followed by 35 cycles (each cycle: 95°C for 40 sec, 55°C for 40 sec, 72°C for 2 min) of amplification, and ending at 72°C for 10 min. The PCR products were analyzed in 1.5% agarose gel, followed by ethidium bromide staining.

The amplified PCR products were ligated into the plasmid pCR2 vector (Original TA cloning Kit, Invitrogen Corporation, Carlsbad, CA) according to the manufacturer’s instruction. The ligation mixture was used to transform the competent Escherichia coli strain DH5α (Gibco BRL, Life Technologies, Gaithersburg, MD) and incubated overnight in LB agar plate containing 100 µg/ml ampicillin and 30 µg/ml X-gal at 37°C. The successful ligation clones in blue white screening were picked-up and cultured overnight in 3 ml LB-Amp broth at 37°C. Plasmid DNA was purified by QIAprep Spin MiniPrep Kit (QIAGEN GmbH, D-40724 Hilden). Sequencing of pre-S1, pre-S2, and S gene was performed with dye terminator cycle sequencing kit (Dye terminator cycle sequencing core kit #402117, Perkin Elmer Cetus Corp., Norwalk, CT) according to the manufacturer’s instruction and sequencing products were analyzed in an ABI 373A sequencer (Perkin Elmer Cetus Corp., Norwalk, CT).

The HBV DNA levels were measured by a Cobas Amplicor HBV monitor (Roche Diagnostic System, Basel, Switzerland), with a detection limit of 300 copies/mL. In this test, 5.82 copies/mL was equal to 1 IU/mL. Genotyping of HBV was performed by PCR restriction fragment length polymorphism of the surface gene of HBV and was further verified by sequencing as previously described [2].

### Statistical Analysis

All statistical analyses were performed using the SPSS 17.0 for Windows (SPSS. Inc., Chicago, IL, USA). _ENREF_18Pearson chi-square analysis or Fisher’s exact test were used to compare categorical variables, while continuous variables were compared using the Mann-Whitney U-test. Cumulative recurrence rate or overall survival rates were estimated by the Kaplan-Meier method and compared using the log-rank test. Variables with statistical significance (*p*<0.05) or proximate to it (*p*<0.1) by univariate analysis underwent multivariate analysis by forward stepwise Cox regression model. A two-tailed *p<*0.05 was considered statistically significant.

## Results

### Baseline Clinical Demographics, Biochemical, Surgical, Virological, and Pathological Data of all Patients

The 333 cases had a median age of 56 years and median tumor size of 4.0 cm. The criteria for the indication of reimbursed antiviral therapy for chronic hepatitis B in Taiwan were as the followings: (1) for cirrhotic patients, serum HBV DNA levels >2000 IU/mL irrespective of serum alanine aminotransferase (ALT) levels; (2) for non-cirrhotic patients, serum ALT levels >80 U/L in addition to serum HBV DNA levels >20000 IU/mL in HBeAg-positive patients and HBVDNA levels >2000 IU/mL in HBeAg-negative patients, respectively. As reimbursed anti-viral therapy was implemented in Taiwan since 2003; therefore, only 62 (18.6%) patients received anti-viral therapy after resection, including 40 with lamivudine, 19 with entecavir, and 3 with pegylated interferon. The demographic characteristics between patients with and those without anti-viral therapy after resection surgery are shown in **Table S2**.

The HBV genotype distribution was one genotype A, 174 genotype B, 139 genotype C, and 19 unclassified. Among the unclassified HBV genotypes, 9 were due to undetectable viral genomes, while the remaining 10 were due to weak signals. Compared to genotype B, patients with genotype C HBV had lower serum albumin levels, lower platelet counts, higher serum ALT and aspartate aminotransferase (AST) levels, higher ICG-15R, and prothrombin time international normalized ratio values **(** **Table 1** **)**. Moreover, prevalence of cirrhosis was higher in patients with genotype C HBV.

**Table 1:** Characteristics of HBV-induced HCC patients who underwent resection surgery.

	All patients (n = 333)	Genotype B (n = 174)	Genotype C (n = 139)	*p*
**Patient Demographics** (continuous variables are expressed as median; 25 and 75 percentiles)				
Age (years)	56; 47–67	56; 44–68.3	57; 48–64	0.692
Sex (male/female) (%)	288/45 (86.5%/13.5%)	153/21 (87.9%/12.1%)	118/21 (84.9%/15.1%)	0.537
Body mass index*	23.5; 21.2–25.6	23.7; 21.2–25.6	23.5; 21.4–25.7	0.598
Albumin (g/dL)*	4.0; 3.8–4.3	4.1; 3.9–4.4	3.9; 3.7–4.2	<0.001
Total bilirubin (mg/dL)*	0.9; 0.7–1.2	0.8; 0.7–1.1	0.9; 0.7–1.2	0.715
ALT (U/L)*	42; 28–66	40; 26–61.5	46; 31.8–73.3	0.017
AST (U/L)*	44; 30–67	41; 29–59.3	52; 33–76	0.007
Alk–P (U/L)*	93; 72–121	90.5; 70.8–121	95; 73–117	0.711
GGT (U/L)*	46; 27–88	41; 24–81.3	53; 28–94.5	0.879
ICG-15R (%)*	10; 6–16	10; 6–15	12; 7–17.5	0.024
Platelet (/mm^3^)*	162000; 120500–212500	167500; 132500–215750	143000; 104000–200000	0.023
PT INR*	1.01; 0.95–1.07	1.00; 0.95–1.05	1.02; 0.96–1.13	0.011
**Viral factors** (continuous variables are expressed as median; 25 and 75 percentiles)				
HBeAg (yes/no) (%)*	34/272(11.1%/88.9%)	11/145(7.1%/92.9%)	23/109(17.4%/82.6%)	0.011
HBVDNA(copies/mL)*	3.90×10^5^; 1.73×10^4^–7.64×10^6^	3.21×10^5^; 2.24×10^4^–4.17×10^6^	1.76×10^5^; 2.45×10^4^–1.39×10^6^	0.390
HBsAg (IU/mL)*	830.0; 287.5–1830.0	935.0; 400.5–1781.3	897.0; 200.8–1959.4	0.241
Pre-core (G1896A) mutation (yes/no) (%)*	194/102 (65.5%/34.5%)	119/46 (72.1%/27.9%)	74/55 (57.4%/42.6%)	0.012
BCP (A1762T, G1764A) mutation (yes/no) (%)*	209/86 (70.8%/29.2%)	96/69 (58.2%/41.8%)	112/16 (87.5%/12.5%)	<0.001
Anti-viral therapy (yes/no) (%)	62/271 (18.6%/81.4%)	39/135 (22.4%/77.6%)	19/120 (13.7%/86.3%)	0.067
**Tumor characteristics** (continuous variables are expressed as median; 25 and 75 percentiles)				
Tumor size (cm)	4.0; 2.5–6.8	4.1; 2.5–6.7	3.5; 2.5–6.3	0.975
Multi-nodularity/single tumor (%)	141/192 (42.3%/57.7%)	74/100 (42.5%/57.5%)	60/79 (43.2%/56.8%)	1.000
Macroscopic venous invasion (yes/no) (%)*	61/271 (18.4%/81.6%)	36/138(20.7%/79.3%)	20/118(14.5%/85.5%)	0.205
AFP (ng/ml)	45.5; 7.4–974.5	31.3; 7.0–996.5	51.1; 8.2–714.8	0.146
Cut margin ≤1/>1 cm (%)*	223/109 (67.2%/32.8%)	125/48 (72.3%/27.7%)	86/53 (61.9%/38.1%)	0.068
**Histo-pathological findings**				
Cirrhosis (yes/no) (%)*	143/180 (44.3%/55.7%)	58/108 (34.9%/65.1%)	76/61 (55.5%/44.5%)	0.001
Edmondson grading (I–II/III–IV) (%)*	214/108 (66.5%/33.5%)	117/50 (70.1%/29.9%)	87/49 (64.0%/36.0%)	0.317
Microscopic venous invasion (yes/no) (%)*	221/111 (66.6%/33.4%)	117/57 (67.2%/32.8%)	92/46 (66.7%/33.3%)	1.000
**Tumor staging**				
BCLC (A/B/C) (%)	182/98/49 (55.3%/29.8%/14.9%)	93/50/30 (53.8%/28.9%/17.3%)	82/40/15 (59.9%/29.2%/10.9%)	0.258

* missing data at the time of resection surgery for this parameter.

Abbreviations: ALT, alanine aminotransferase; AST, aspartate aminotransferase; Alk-P, alkaline phosphatase; GGT, gamma-glutamyltransferase; ICG-15R, indocyanine green retention rate at 15 minutes; PT, prothrombin time; INR, international normalized ratio; HBsAg, hepatitis B surface antigen; BCP, basal core promoter; BCLC, the Barcelona-Clinic Liver Cancer.

*p* value: comparison between genotype B and C.

Regarding viral factors, patients with genotype C HBV had a higher prevalence of positive HBeAg in sera and BCP mutation, and lower frequency of pre-core mutation (G1896A) than those with genotype B, but serum HBV DNA and HBsAg levels were comparable between these two groups. Tumor characteristics were not significantly different between the two groups.

### Factors Associated with Poor Overall Survival and Recurrence after Resection

After a median follow-up of 45.9 months (25^th^-to-75^th^ percentile range, 22.4–78.9 months), 165 patients died. The overall cumulative survival rates at 1, 3, and 5 years were 86.7%, 69.3%, and 55.4%, respectively. Moreover, 208 patients had tumor recurrence. The cumulative recurrence-free rates at 1, 3, and 5 years were 66.7%, 44.7%, and 35.3%, respectively.

**Figure 1:**
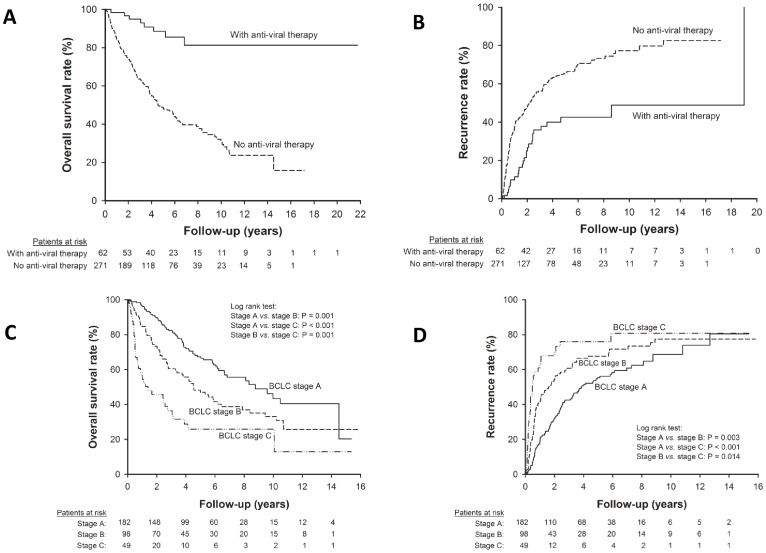
The impact of anti-viral therapy and tumor stage on post-operative prognosis. Patients who received anti-viral therapy after resection had (**A**) higher overall survival rate (*p*<0.001) and (**B**) lower recurrence rate (*p*<0.001) than those who did not receive anti-viral therapy. Moreover, patients in BCLC stage C HCC had lower overall survival rate (**C**) and higher recurrence rate (**D**) than those in BCLC stage A or B.

Univariate analysis of risk factors associated with overall survival and recurrence were shown in **Table S3 and Table S4**, respectively. By multivariate analysis, ICG-15R >10%, alkaline phosphatase (Alk-P) >100 U/L, macroscopic venous invasion, microscopic venous invasion, and the absence of anti-viral therapy were significant risk factors predictive of poor overall survival **(Table S5)**. Furthermore, ICG-15R >10%, gamma-glutamyltransferase (GGT) >60 U/L, macroscopic venous invasion, microscopic venous invasion, and the absence of anti-viral therapy were significant risk factors for tumor recurrence after resection **(** **Table 2** **)**. Consequently, anti-viral therapy was a critical factor for determining post-operative prognosis both in terms of overall survival and recurrence **(** **Figure 1** **)**.

**Table 2:** Multivariate analysis of factors associated with recurrence after resection for HCC.

Variable	Hazard ratio (95% confidence interval)	Standard error		*p*
**All patients (n = 333)**				
ICG-15R >10%	1.386 (1.036–1.854)	0.148	0.028	
GGT >60 U/L	1.628 (1.210–2.190)	0.151	0.001	
Macroscopic venous invasion	2.375 (1.650–3.425)	0.186	<0.001	
Microscopic venous invasion	1.499 (1.080–2.083)	0.167	0.016	
Without anti-viral therapy	2.296 (1.451–3.632)	0.234	<0.001	
**Patients without antiviral therapy after resection (n = 271)**				
HBV DNA >10^6^ copies/mL	1.428 (1.047–1.947)	0.158		0.024
GGT >60 U/L	1.700 (1.239–2.331)	0.194		0.001
Macroscopic venous invasion	2.387 (1.634–3.497)	0.180		<0.001
Microscopic venous invasion	1.594 (1.121–2.273)	0.195		0.010
**Patients with available complete HBs gene sequence data but without anti-viral therapy (n = 216)**				
Multi-nodularity	1.693 (1.115–2.571)	0.213		0.013
Macroscopic venous invasion	2.415 (1.418–4.098)	0.271		0.001
Cirrhosis	1.544 (1.047–2.279)	0.198		0.029
Edmondson stage III or IV	1.526 (1.029–2.263)	0.201		0.036
Pre-S deletion	1.564 (1.057–2.314)	0.200		0.025

Abbreviations: ICG-15R, indocyanine green retention rate at 15 minutes; GGT, gamma-glutamyltransferase.

### The effect of Anti-viral Therapy Stratified by the Barcelona-Clinic Liver Cancer (BCLC) Stage and viral Factors

**Figure 2:**
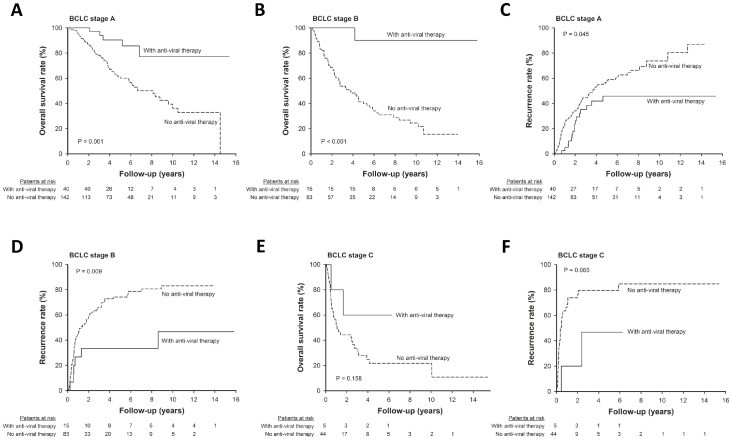
The effect of anti-viral therapy on post-operative prognosis stratified by tumor stage. Among patients with early stage HCC, those who received anti-viral therapy after resection had higher overall survival rate (**A**, BCLC stage A, *p* = 0.001; **B**, BCLC stage B, *p*<0.001) and lower recurrence rate (**C**, BCLC stage A, *p* = 0.045; **D**, BCLC stage B, *p* = 0.009) than their counterpart. For patients with BCLC stage C HCC, the overall survival rate were comparable between those with and without antiviral therapy (**E**, p = 0.158); but patients receiving anti-viral therapy after resection had a trend of lower recurrence rate than those who did not receive anti-viral therapy (**F**, p = 0.065).

As the demographic characteristics were not perfectly match between patients with and those without antiviral therapy after resection. Beside multivariate analysis, we further assessed the efficacy of anti-viral therapy stratified by tumor stage and viral factors to diminish the impact of confounding factors on prognosis. Patients with BCLC tumor stage C HCC had significantly lower overall survival rate and higher recurrence rate than those with stage A or B **(** **Figure 1** **)**. Moreover, anti-viral therapy could improve the post-operative prognosis both in terms of overall survival and recurrence in patients with early stage HCC, but the effect was less apparent in those with BCLC stage C **(** **Figure 2** **)**.

**Figure 3:**
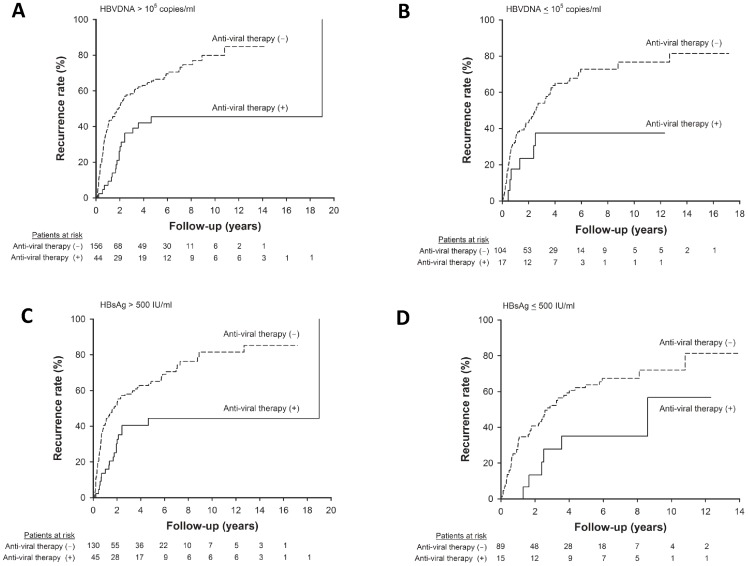
The impact of anti-viral therapy on post-operative recurrence stratified by viral factors. Patients who received anti-viral therapy after resection had significantly lower recurrence rate both in the setting of serum HBV DNA levels >10^5^ copies/mL (**A**, *p*<0.001) and ≤10^5^ copies/mL (**B**, *p* = 0.038). (**C**) Among patients with serum HBsAg >500 IU/mL, anti-viral therapy was associated with lower recurrence rate (*p* = 0.001). (**D**) In patients with serum HBsAg ≤500 IU/mL, the recurrence rates were also lower in patients receiving antiviral therapy after resection surgery (*p* = 0.037).

Regarding viral factors, anti-viral therapy could reduce post-operative recurrence both in the setting of high and low serum HBV DNA levels **(** **Figure 3A–B** **)**. Similarly, the recurrence rates were both lower in patients receiving anti-viral therapy than their counterpart irrespective of their serum HBsAg levels **(** **Figure 3C–D** **)**.

### Factors Associated with Overall Survival and Recurrence in HCC Patients without Anti-viral Therapy

As anti-viral therapy might confound the impact of viral factors, to eliminate this bias, 62 patients who received anti-viral therapy after resection surgery were excluded and the remaining 271 patients were further analyzed for prognostic factors.

By multivariate analysis, serum HBVDNA levels >10^6^ copies/mL, GGT >60 U/L, macroscopic venous invasion, and microscopic venous invasion were significant risk factors predictive of tumor recurrence after resection **(** **Table 2** **)**. The result of multivariate analysis of significant risk factors associated with poor overall survival is shown in **Table S5**.

### Factors Associated with Poor Prognosis in the HCC Sub-group with Available Complete HBs Gene Sequence Data but without Anti-viral Therapy

Among the 216 patients with sufficient serum samples for complete HBs gene sequencing, 73 had the pre-S deletion HBV mutants, including 15 with the pre-S1 deletion mutant only, 43 with the pre-S2 deletion mutant only, and 15 with both.

Of the 58 patients with the pre-S2 deletion mutants HBV, 25 (43.1%) had the deletion that ended at amino acid 142 of the pre-S region. Proline was the preserved amino acid on this site in all of these mutants.

The demographic characteristics, including tumor factors, liver functional reserve and virological factors (genotype, serum HBVDNA and HBsAg levels, proportion of BCP and pre-core mutations) were similar between patients with pre-S1 deletion mutants and those with pre-S2 deletion mutants.

Comparing the demographic characteristics between patients with and those without the pre-S deletion HBV mutants, those with the pre-S deletion mutants had a higher rate of liver cirrhosis on the non-tumor part, lower serum albumin levels, higher GGT levels, and a trend of higher ALT level **(** **Table 3** **)**. These imply that HCC patients with the pre-S deletion mutants of HBV had more advanced hepatic fibrosis and poorer liver functional reserve. Regarding viral factors, the proportion of positive HBeAg in sera and genotype C were higher in patients infected with the pre-S deletion HBV mutants than those without the pre-S deletion mutants. Moreover, patients with the pre-S deletion had a trend of higher serum HBVDNA levels (*p* = 0.055). Tumor factors were comparable between the two groups.

**Table 3:** Comparison of HCC patients with and without the pre-S deletion mutants of HBV.

	Without Pre-S deletion (n = 143)	With Pre-S deletion (n = 73)	*p*
Age (years)	58; 46–69	54; 46–64	0.500
Sex (male/female) (%)	123/20 (86/0%/14.0%)	65/8 (89.0%/11.0%)	0.680
Albumin (g/dL)*	4.1;3.9–4.4	4.0; 3.7–4.2	0.018
Total bilirubin (mg/dL)*	0.9; 0.7–1.2	0.9; 0.7–1.3	0.847
ALT (U/L)*	42; 27.5–65.6	49; 39–74	0.084
AST (U/L)*	42.5; 28–62.5	51; 33–79.5	0.104
Alk-P (U/L)*	84; 70.5–107.0	97; 72.5–118.5	0.359
GGT (U/L)*	38; 26–70	53; 23–133	0.008
ICG-15R (%)*	11; 6–16	10; 7–17	0.735
Platelet (/mm^3^)*	156000; 125000–199000	148000; 98000–209000	0.849
PT INR*	1.01; 0.96–1.06	1.00; 0.94–1.12	0.571
HBVDNA(copies/mL)*	4.85×10^5^; 3.67×10^4^–1.08×10^7^	1.61×10^6^; 9.95×10^4^–1.51×10^7^	0.055
HBsAg (IU/mL)*	896.3; 339.5–1732.4	1127.5; 405–2101	0.297
HBeAg (yes/no) (%) *	10/119(7.8%/92.2%)	15/53(22.1%/77.9%)	0.008
Genotype B/C (%)	98/44 (69.0%/31.0%)	33/40 (45.2%/54.8%)	0.001
Pre-core (G1896A) mutation (yes/no) (%)*	88/42 (67.7%/32.3%)	40/28 (58.8%/41.2%)	0.279
BCP (A1762T, G1764A) mutation (yes/no) (%)*	83/47 (63.8%/36.2%)	52/16 (76.5%/23.5%)	0.099
Tumor size (cm)	3.4; 2.5–6.0	3.0; 2.1–6.0	0.191
Multi-nodularity/single tumor (%)	57/86 (39.9%/60.1%)	25/48 (34.2%/65.8%)	0.512
Macroscopic venous invasion (yes/no) (%)*	21/121 (14.8%/85.2%)	12/61 (16.4%/83.6%)	0.906
AFP (ng/ml)	20.8; 6.7–752.3	50.6; 12.1–803.0	0.343
Cut margin ≤1/>1 cm (%)*	101/42 (70.6%/29.4%)	45/27 (62.5%/37.5%)	0.294
Cirrhosis (yes/no) (%)*	51/86 (37.2%/62.8%)	40/30 (57.1%/42.9%)	0.010
Edmondson grading (I–II/III–IV) (%)*	47/91 (34.1%/65.9%)	18/53 (25.4%/74.6%)	0.258
Microscopic venous invasion (yes/no) (%)*	93/49 (65.5%/34.5%)	47/26 (64.4%/35.6%)	0.992
BCLC (A/B/C) (%)	84/40/14 (59.6%/28.4%/12.1%)	45/16/11 (62.5%/22.2%/15.3%)	0.566

* missing data at the time of resection surgery for this parameter.

Continuous variables are expressed as median; 25 and 75 percentiles.

Abbreviations: ALT, alanine aminotransferase; AST, aspartate aminotransferase; Alk-P, alkaline phosphatase; GGT, gamma-glutamyltransferase; ICG-15R, indocyanine green retention rate at 15 minutes; PT, prothrombin time; INR, international normalized ratio; HBsAg, hepatitis B surface antigen; BCP, basal core promoter; BCLC, the Barcelona-Clinic Liver Cancer.

Univariate analysis of risk factors that correlated with overall survival and recurrence were shown in **Table S6 and Table S7**, respectively. By multivariate analysis, multi-nodularity, presence of macroscopic venous invasion, cirrhosis, advanced tumor cell differentiation, and pre-S deletion were the significant risk factors predictive of post-resection recurrence **(** **Table 2** **)**. Besides, the recurrence-free survival rates were comparable between patients with pre-S1 deletion mutants and those with pre-S2 deletion mutants.

### Impact of Pre-S deletion on Post-operative Recurrence Stratified by Serum HBV DNA and HBsAg Levels

**Figure 4:**
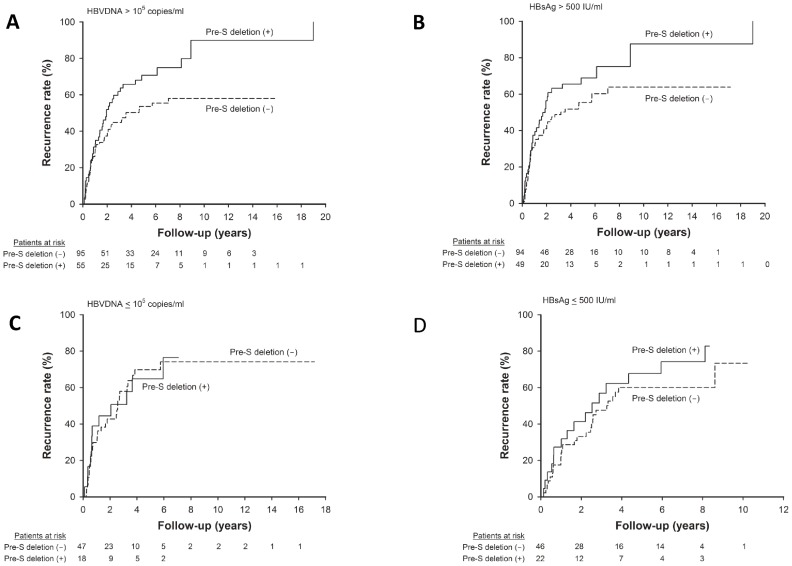
Comparison of post-resection recurrence between patients with and those without pre-S deletion mutants stratified by serum HBVDNA and HBsAg levels. (**A**) Among patients with serum HBVDNA >10^5^ copies/mL, the presence of pre-S deletion correlated with a higher rate of post-resection recurrence than patients without the pre-S deletion (*p* = 0.028). (**B**) Among patients with serum HBsAg >500 IU/mL, pre-S deletion was associated with higher recurrence rate after resection (*p* = 0.047). (**C**) In patients with serum HBVDNA ≤10^5^ copies/mL, the recurrence rates were comparable between patients with and those without pre-S deletion (*p* = 0.926). (**D**) In patients with serum HBsAg ≤500 IU/mL, the recurrence rates were similar between patients with and those without pre-S deletion (*p* = 0.253).

The effects of pre-S deletion in post-operative recurrence as stratified by serum HBV viral load and HBsAg levels were assessed. For patients with higher serum HBV DNA (>10^5^ copies/mL) or higher HBsAg level (>500 IU/mL), the presence of pre-S deletion was significantly associated with a higher rate of recurrence **(** **Figure 4** **)**. However, for those with low viral loads or lower HBsAg levels, recurrence rates were comparable between patients with and those without pre-S deletion.

## Discussion

For patients with HBV-induced HCC, ongoing HBV viral replication may initiate tumor recurrence after resection through several mechanisms [2], [14]. Firstly, it can induce persistent hepatic inflammation, oxidative stress, and fibrosis, which in turn leads to hepatocarcinogenesis. However, it may need longer time to accumulate sufficient mutations for tumor recurrence simply by chronic inflammation. Our previous study shows that the grade of hepatic inflammation is crucial for the development of late recurrence (recurrence occurring after two years of resection surgery) [2]. Second, it may also have direct mutagenic effects through the integration of the HBV DNA into the host genome, initiation of oncogenes mediated by prolonged expression of viral proteins, and inhibition of tumor suppressor genes like p53 [24], [25]. One recent genome-wide survey for HBV-induced HCC demonstrates that HBV integration occurs more frequently in tumors (86.4%) than in the adjacent liver tissues (30.7%). [25] Moreover, the HBV integration breakpoints seem to be associated with increased copy-number variations and chromosome instability.

Because anti-viral therapy can suppress HBV replication, ameliorate liver inflammation and fibrosis, and improve liver functional reserve, meta-analysis of previous studies reveals that anti-viral therapy improves the overall survival of HCC patients after curative therapy [26]. However, its role in reducing recurrence rate is still controversial. This may be due to differences in demographic characteristics, liver functional reserve, and tumor factors in previous reports. The patient numbers are also relatively smaller in these studies [26], [27]. A recent study using National Health Insurance Research Dataset suggested that antiviral therapy might reduce postoperative HCC recurrence [28]. However, the dataset did not provide detail analysis of HBV loads, HBsAg levels and HBV mutations that are closely linked to the development of HCC [9], [10], [29], [30].

In this cohort, for those without anti-viral therapy after resection, higher serum HBV DNA level was an important risk factor associated with recurrence. Of note, anti-viral therapy can improve the overall survival rate and reduce recurrence after resection even in patients with high viral loads and HBsAg levels and the latter two factors were not significantly associated with recurrence in the presence of antiviral therapy. Antiviral therapy should be recommended as tertiary preventive measure for the reduction of postoperative HCC recurrence in patients with HBV-related HCC.However, the impact of anti-viral therapy on improving post-operative outcomes was less significant in patients with BCLC stage C. It may be attributed to that tumor factors dominate the prognoses in patients with advanced stage HCC even after curative therapies, thereby diminish the effect of anti-viral therapy in improving the “field factors” of non-tumor part [2], [27].

The pre-S region of HBV is crucial in mediating the attachment of the virus to host hepatocytes. Moreover, it is important for interacting with the host immune responses [17]. It has been reported that the proportions of pre-S deletion mutants are significantly higher in patients with HBV-related HCC than in those with inactive HBV carrier status, implying that these mutants may be associated with HCC carcinogenesis [17], [19]. Previous studies show that the deletion mutants in the pre-S region can cause the accumulation of large surface proteins in the endoplasmic reticulum (ER) and induce ER stress, which in turn provokes mutagenesis through the generation of oxidative stress, cyclooxygenase-2 expression, and DNA damages [17], [31]. One recent study further demonstrates that the ER stress induced by pre-S mutants leads to disturbances in cyclin A expression and centrosome over-duplication, thereby causing chromosome instability and hepatocarcinogenesis [31]. However, there have been few reports about the influence of pre-S deletion mutants on postoperative HCC recurrence. Our current study indicates that for patients with HBV-related HCC, the presence of pre-S deletion mutants is an important factor for predicting post-operative HCC recurrence. This implies the critical role of pre-S deletion mutants of HBV both in hepatocarcinogenesis and tumor recurrence.

In this study, patients with pre-S deletion mutants have a higher proportion of positive HBeAg in sera and a trend of higher HBV DNA levels than their counterpart in the present cohort. As most of the deletion regions encompassed T and B cell epitopes, it is likely that pre-S deletion HBV mutants may escape more easily from host immune clearance, resulting in higher viral replication than those without pre-S deletion [19]. Of note, the large number of patients in this study which provided subgroup analysis firstly reported that the presence of pre-S deletion mutants correlates to a higher rate of tumor recurrence only in patients with serum HBV DNA levels >10^5^ copies/mL or HBsAg >500 IU/mL. In contrast, the recurrence rates are similar between patients with and those without pre-S deletion in patients with low viral loads. Moreover, for patients receiving antiviral therapy after resection, the prognoses were comparable between patients with and those without pre-S deletion mutants **(Figure S1)**. This implies that the ER and oxidative stresses caused by the accumulation of large surface protein may be more apparent in the setting of high HBV DNA levels. In other words, the hepatocarcinogenesis and tumor recurrence induced by pre-S deletion mutants may be based on active HBV viral replication, the accumulation of oxidative stress, and subsequent chromosomal instability [31].

In conclusion, on-going viral replication and the presence of pre-S deletion are both crucial predictors of post-operative HCC recurrence. Anti-viral therapy can reduce post-operative recurrence and improve prognosis, especially for those with early stage HCC.

## Supporting Information

Figure S1 **The impact of pre-S deletion mutants on post-operative recurrence for patients receiving anti-viral therapy.** The recurrence rate after resection surgery were comparable between patients with and those without pre-S deletion mutants (*p* = 0.161).
(TIF)

Table S1 **Comparison of demographic characteristics between patients with and those without detail virological data of hepatitis B virus.**
(DOCX)

Table S2 **Comparison of demographic characteristics between patients with and those without antiviral therapy after resection surgery.**
(DOCX)

Table S3 **Univariate analysis of factors associated with overall survival after resection for hepatocellular carcinoma.**
(DOCX)

Table S4 **Univariate analysis of factors associated with recurrence after resection for hepatocellular carcinoma.**
(DOCX)

Table S5 **Multivariate analysis of factors associated with overall survival after resection for HCC.**
(DOCX)

Table S6 **Univariate analysis of factors associated with overall survival after resection for hepatocellular carcinoma with available complete HBs gene sequence data.**
(DOCX)

Table S7 **Univariate analysis of factors associated with recurrence after resection for hepatocellular carcinoma with available complete HBs gene sequence data.**
(DOCX)
