# Supplementary figures and images for: The Influence of Hepatitis B Viral Load and Pre-S Deletion Mutations on Post-Operative Recurrence of Hepatocellular Carcinoma and the Tertiary Preventive Effects by Anti-Viral Therapy
Source: PLoS One. 2013 Jun 21;8(6):e66457. doi: 10.1371/journal.pone.0066457 (PMC3689837; doi:10.1371/journal.pone.0066457)

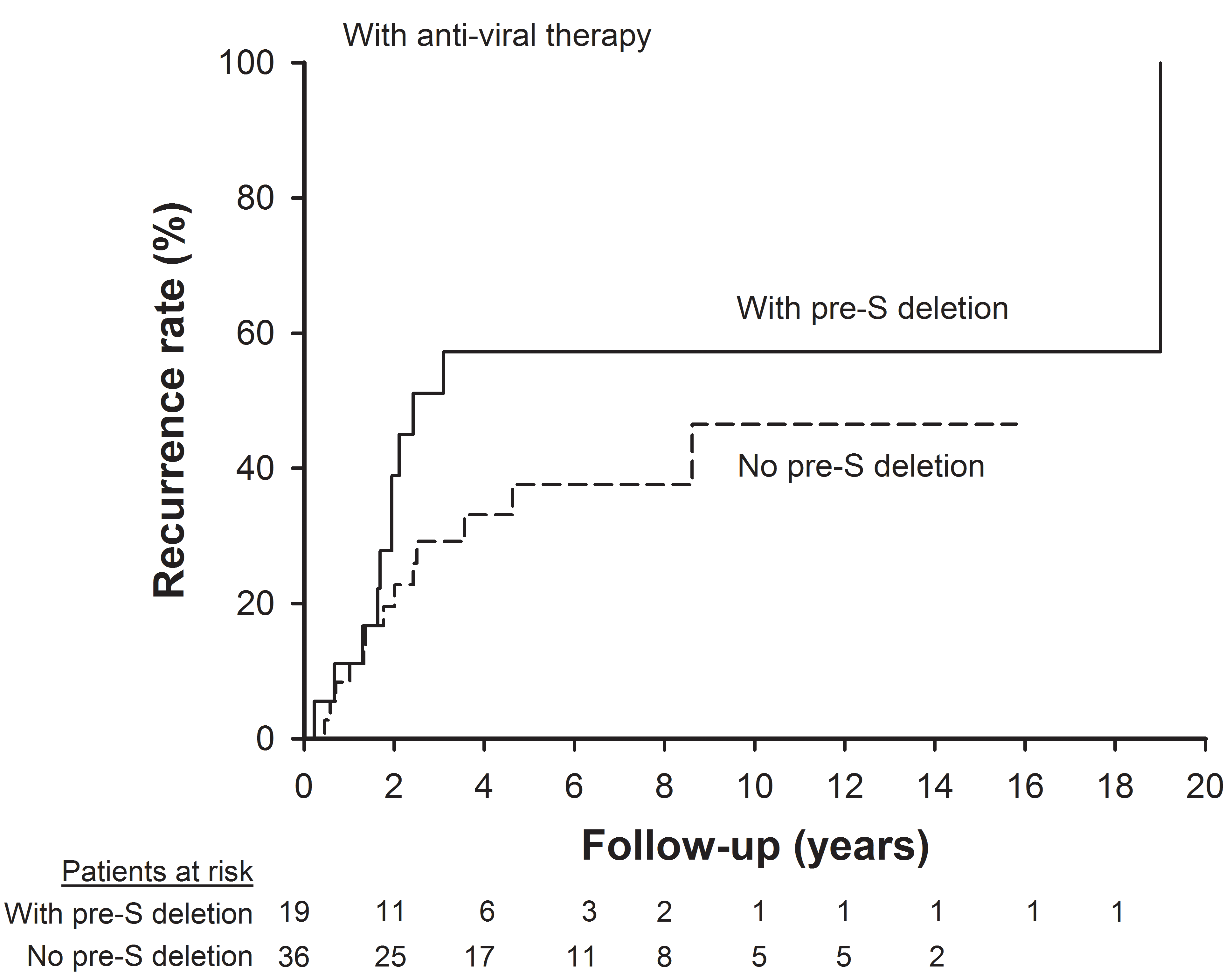

Supplement: Figure S1 — The impact of pre-S deletion mutants on post-operative recurrence for patients receiving anti-viral therapy. The recurrence rate after resection surgery were comparable between patients with and those without pre-S deletion mutants (p = 0.161). (TIF) [file pone.0066457.s001.tif]
