# Supplementary material for: The Influence of Hepatitis B Viral Load and Pre-S Deletion Mutations on Post-Operative Recurrence of Hepatocellular Carcinoma and the Tertiary Preventive Effects by Anti-Viral Therapy
Source: PLoS One. 2013 Jun 21;8(6):e66457. doi: 10.1371/journal.pone.0066457 (PMC3689837; doi:10.1371/journal.pone.0066457)
Supplement: Table S1 — Comparison of demographic characteristics between patients with and those without detail virological data of hepatitis B virus. (DOCX) [file pone.0066457.s002.docx]

**Table S1. Comparison of demographic characteristics between patients with and those without detail virological data of hepatitis B virus**

|  | **Patients with detail virological data**  **(n=333)** | **Patients without detail virological data**  **(n=274)** | ***p*** |
| --- | --- | --- | --- |
| Age (years) | 56; 47-67 | 55.5; 46-68.3 | 0.939 |
| Sex (male/female) (%) | 288/45 (86.5%/13.5%) | 227/47 (82.8%/17.2%) | 0.258 |
| Albumin (g/dL)* | 4.0; 3.8-4.3 | 4.1; 3.8-4.3 | 0.992 |
| Total bilirubin (mg/dL)* | 0.9; 0.7-1.2 | 0.9; 0.6-1.1 | 0.400 |
| ALT (U/L)* | 42; 28-66 | 44; 30-66 | 0.904 |
| AST (U/L)* | 44; 30-67 | 46; 35-70 | 0.815 |
| Alk-P (U/L)* | 93; 72-121 | 94; 71-132 | 0.272 |
| GGT (U/L)* | 46; 27-88 | 55; 34-102 | 0.261 |
| ICG-15R (%)* | 10; 6-16 | 10; 6-15 | 0.327 |
| Platelet (/mm^3^)* | 162000; 120500-212500 | 161500; 111750-221000 | 0.455 |
| PT INR* | 1.01; 0.95-1.07 | 1.00; 1.00-1.09 | 0.236 |
| Tumor size (cm) | 4.0; 2.5-6.8 | 4.6; 2.7-8.5 | 0.018 |
| Multi-nodularity/single tumor (%) | 141/192 (42.3%/57.7%) | 129/144 (47.3%/52.7%) | 0.259 |
| Macroscopic venous invasion (yes/no) (%)* | 61/271 (18.4%/81.6%) | 57/215 (21.0%/79.0%) | 0.488 |
| AFP (ng/ml) | 45.5; 7.4-974.5 | 48.5; 9.3-651.8 | 0.629 |
| Cut margin ≤1/>1 cm (%)* | 223/109 (67.2%/32.8%) | 195/78 (71.4%/28.6%) | 0.298 |
| Cirrhosis (yes/no) (%)* | 143/180 (44.3%/55.7%) | 112/154 (42.1%/57.9%) | 0.657 |
| Edmondson grading (I-II/III-IV) (%)* | 214/108 (66.5%/33.5%) | 187/79 (70.3%/29.7%) | 0.365 |
| Microscopic venous invasion (yes/no) (%)* | 221/111 (66.6%/33.4%) | 179/95 (65.3%/34.7%) | 0.815 |

Continuous variables are expressed as median; 25 and 75 percentiles.

*missing data at the time of resection surgery for this parameter.

Abbreviations: ALT, alanine aminotransferase; AST, aspartate aminotransferase; Alk-P, alkaline phosphatase; GGT, gamma-glutamyltransferase; ICG-15R, indocyanine green retention rate at 15 minutes; PT, prothrombin time; INR, international normalized ratio.
