# Supplementary material for: The Influence of Hepatitis B Viral Load and Pre-S Deletion Mutations on Post-Operative Recurrence of Hepatocellular Carcinoma and the Tertiary Preventive Effects by Anti-Viral Therapy
Source: PLoS One. 2013 Jun 21;8(6):e66457. doi: 10.1371/journal.pone.0066457 (PMC3689837; doi:10.1371/journal.pone.0066457)
Supplement: Table S2 — Comparison of demographic characteristics between patients with and those without antiviral therapy after resection surgery. (DOCX) [file pone.0066457.s003.docx]

**Table S2. Comparison of demographic characteristics between patients with and those without antiviral therapy after resection surgery.**

|  | **Patients with antiviral therapy**  **(n=62)** | **Patients without antiviral therapy**  **(n=271)** | ***p*** |
| --- | --- | --- | --- |
| Age (years) | 52; 43.8-60 | 58; 48-68 | 0.014 |
| Sex (male/female) (%) | 56/6 (90.3%/9.7%) | 232/39 (85.6%/14.4%) | 0.439 |
| Albumin (g/dL)* | 4.1; 3.9-4.3 | 4.0; 3.8-4.3 | 0.091 |
| Total bilirubin (mg/dL)* | 0.9; 0.6-1.3 | 0.9; 0.7-1.2 | 0.614 |
| ALT (U/L)* | 45; 28.8-64.3 | 42; 28-62 | 0.208 |
| Alk-P (U/L)* | 85; 70-102.5 | 95; 73-123.5 | 0.061 |
| GGT (U/L)* | 32; 22-61 | 49; 28-94 | 0.837 |
| ICG-15R (%)* | 9; 5-16 | 11; 6-16 | 0.452 |
| Platelet (/mm^3^)* | 164500; 134500-199250 | 161000; 116000-216000 | 0.551 |
| HBVDNA(copies/mL)* | 7.85 x 10^5^;  6.70 x 10^4^-1.42 x 10^7^ | 3.03 x 10^5^;  1.23 x 10^4^-6.76 x 10^7^ | 0.207 |
| HBsAg (IU/mL)* | 1121.1; 492.2-2415.0 | 704.0; 240.0-1685.0 | 0.743 |
| HBeAg (yes/no) (%) * | 9/51 (15.0%/85.0%) | 25/221 (10.2%/89.8%) | 0.401 |
| Genotype A/B/C (%) | 0/39/19 (0%/67.2%/32.8%) | 1/135/120 (0.4%/52.7%/46.9%) | 0.125 |
| Pre-core (G1896A) mutation (yes/no) (%)* | 38/16 (70.4%/29.6%) | 156/86 (64.5%/35.5%) | 0.504 |
| BCP (A1762T, G1764A) mutation (yes/no) (%)* | 36/18 (66.7%/33.3%) | 173/68 (71.8%/28.2%) | 0.560 |
| Tumor size (cm) | 2.7; 2.0-5.3 | 4.2; 2.5-7.4 | 0.016 |
| Multi-nodularity/single tumor (%) | 14/48 (22.6%/77.4%) | 127/144 (46.9%/53.1%) | 0.001 |
| Macroscopic venous invasion (yes/no) (%)* | 7/55 (11.3%/88.7%) | 54/216 (20.0%/80.0%) | 0.157 |
| AFP (ng/ml) | 37.4; 7.2-570.0 | 48.0; 7.6-1018.5 | 0.444 |
| Cirrhosis (yes/no) (%)* | 23/38 (37.7%/62.3%) | 120/142 (45.8%/54.2%) | 0.316 |
| Edmondson grading (I-II/III-IV) (%)* | 50/11 (82.0%/18.0%) | 164/97 (62.8%/37.2%) | 0.007 |
| Microscopic venous invasion (yes/no) (%)* | 32/29 (52.5%/47.5%) | 189/82 (69.7%/30.3%) | 0.015 |
| BCLC (A/B/C) (%) | 40/15/5 (66.7%/25.0%/8.3%) | 142/83/44 (52.8%/30.9%/16.3%) | 0.111 |

Continuous variables are expressed as median; 25 and 75 percentiles.

*missing data at the time of resection surgery for this parameter.

Abbreviations: ALT, alanine aminotransferase; AST, aspartate aminotransferase; Alk-P, alkaline phosphatase; GGT, gamma-glutamyltransferase; ICG-15R, indocyanine green retention rate at 15 minutes; PT, prothrombin time; INR, international normalized ratio; BCLC, the Barcelona-Clinic Liver Cancer
