# Supplementary material for: The Influence of Hepatitis B Viral Load and Pre-S Deletion Mutations on Post-Operative Recurrence of Hepatocellular Carcinoma and the Tertiary Preventive Effects by Anti-Viral Therapy
Source: PLoS One. 2013 Jun 21;8(6):e66457. doi: 10.1371/journal.pone.0066457 (PMC3689837; doi:10.1371/journal.pone.0066457)
Supplement: Table S3 — Univariate analysis of factors associated with overall survival after resection for hepatocellular carcinoma. (DOCX) [file pone.0066457.s004.docx]

**Table S3. Univariate analysis of factors associated with overall survival after resection for hepatocellular carcinoma**

| **Variable** | | | **Number** | **Median survival months (95% CI)** | **Hazard ratio (95% CI)** | ***p*** |
| --- | --- | --- | --- | --- | --- | --- |
| Age > 60 / ≤ 60 y/o | | | 131/201 | 50.6 (41.0-60.2)/  115.1 (78.9-151.3) | 1.743  (1.283-2.367) | <0.001 |
| Sex Female/Male | | | 45/288 | 128.3 (25.7-230.9)/  70.4 (53.7-87.1) | 0.690  (0.422-1.126) | 0.137 |
| Albumin ≤ 4 / > 4 g/dL | | | 165/163 | 46.1 (36.0-56.2)/  125.9 (80.4-171.4) | 1.843  (1.345-2.526) | <0.001 |
| Bilirubin > 1.6 / ≤ 1.6 mg/dL | | | 20/312 | 81.9 (46.3-117.5)/  71.2 (48.0-94.4) | 0.855  (0.450-1.623) | 0.631 |
| ALT >40 / ≤ 40 U/L | | | 178/154 | 70.4 (52.0-88.8)/  79.7 (42.8-116.6) | 1.087  (0.799-1.479) | 0.594 |
| Alk-P >100 / ≤ 100 U/L | | | 132/199 | 41.2 (26.1-56.3)/  100.9 (73.1-128.7) | 1.892  (1.392-2.571) | <0.001 |
| GGT >60 / ≤ 60 U/L | | | 131/197 | 46.7 (35.1-58.3)/  113.5 (92.6-134.4) | 1.843  (1.356-2.506) | <0.001 |
| Platelet ≤ 10^5^ / > 10^5^ /mm^3^ | | | 51/262 | 60.3 (34.9-85.7)/  74.2 (45.5-102.9) | 1.149  (0.776-1.702) | 0.488 |
| ICG-15R > 10% / ≤ 10% | | | 160/170 | 52.3 (34.2-70.4)/  120.9 (80.1-161.7) | 1.707  (1.250-2.330) | 0.001 |
| HBeAg (Y/N) | | | 34/272 | 50.3(24.4-76.2)/  74.2(52.8-95.6) | 0.738  (0.469-1.161) | 0.187 |
| HBV genotype C/B | | | 138/174 | 72.8 (52.2-93.4)/  69.9 (23.6-116.2) | 1.120  (0.821-1.529) | 0.475 |
| HBV DNA >10^6^ / ≤10^6^ copies/mL | | | 136/184 | 70.4 (29.6-111.2)/  75.1 (55.8-94.4) | 0.980  (0.715-1.344) | 0.901 |
| HBsAg > 1000 / ≤1000 IU/mL | | | 121/157 | 79.7 (45.1-114.3)/  100.0 (64.0-136.0) | 1.142  (0.803-1.624) | 0.459 |
| G1896A mutation (Y/N) | | | 193/102 | 94.5 (61.6-127.4)/  54.5 (33.7-75.3) | 0.752  (0.544-1.040) | 0.085 |
| A1762T/G1764A mutation (Y/N) | | | 209/85 | 71.2 (56.8-85.6)/  70.9 (0-165.9) | 1.040  (0.722-1.499) | 0.833 |
| Anti-viral therapy (N/Y) | | | 271/62 | 54.5 (40.0-69.0)/  NA | 5.938  (2.915-12.094) | <0.001 |
| Tumor size > 5cm / ≤ 5cm | | | 122/210 | 37.5 (22.6-52.4)/  100.0 (68.0-132.0) | 2.001  (1.472-2.721) | <0.001 |
| Multi-nodularity (Y/N) | | | 141/191 | 45.7 (33.6-57.8)/  119.8 (97.9-141.7) | 2.182  (1.603-2.972) | <0.001 |
| Macroscopic venous invasion (Y/N) | | | 61/271 | 16.8 (0-36.5)/  94.5 (67.3-121.7) | 2.809  (1.976-3.984) | <0.001 |
| Cut margin ≤ 1cm/ >1cm | 223/108 | 60.3 (45.9-74.7)/  113.5 (89.3-137.7) | 1.520  (1.079-2.141) | 0.017 |  |  |
| AFP >20 / ≤ 20 ng/ml | | 192/136 | 69.7 (48.1-91.3)/  81.9 (48.4-115.4) | 1.308  (0.951-1.799) | 0.099 |  |
| Microscopic venous invasion (Y/N) | | 221/110 | 52.3 (40.6-64.0)/  119.8 (103.7-135.9) | 2.203  (1.527-3.185) | <0.001 |  |
| Cirrhosis (Y/N) | | 143/179 | 54.2 (40.9-67.5)/  122.5 (82.9-162.1) | 1.706  (1.248-2.331) | 0.001 |  |
| Edmonson stage III or IV/ I or II | | 214/108 | 79.9 (54.0-105.8)/  64.0 (38.3-89.7) | 1.369  (0.997-1.879) | 0.052 |  |
| BCLC stage B or C/ A | | 147/182 | 40.6 (26.8-54.4)/  105.3 (72.6-138.0) | 2.172  (1.593-2.963) | <0.001 |  |

Abbreviations: ALT, alanine aminotransferase; AST, aspartate aminotransferase; Alk-P, alkaline phosphatase; GGT, gamma-glutamyltransferase; ICG-15R, indocyanine green retention rate at 15 minutes; HBsAg, hepatitis B surface antigen; BCP, basal core promoter; BCLC, the Barcelona-Clinic Liver Cancer; NA, not applicable; N: no; Y: yes.
