# Supplementary material for: The Influence of Hepatitis B Viral Load and Pre-S Deletion Mutations on Post-Operative Recurrence of Hepatocellular Carcinoma and the Tertiary Preventive Effects by Anti-Viral Therapy
Source: PLoS One. 2013 Jun 21;8(6):e66457. doi: 10.1371/journal.pone.0066457 (PMC3689837; doi:10.1371/journal.pone.0066457)
Supplement: Table S4 — Univariate analysis of factors associated with recurrence after resection for hepatocellular carcinoma. (DOCX) [file pone.0066457.s005.docx]

**Table S4. Univariate analysis of factors associated with recurrence after resection for hepatocellular carcinoma**

| **Variable** | **Number** | **Median recurrence months (95% CI)** | **Hazard ratio (95% CI)** | ***p*** |
| --- | --- | --- | --- | --- |
| Age > 60 / ≤ 60 y/o | 131/201 | 25.0 (10.4-39.7)/  32.6 (16.4-48.8) | 1.294  (0.982-1.705) | 0.067 |
| Sex Female/Male | 45/288 | 32.2 (0-66.4)/  29.5 (21.4-37.6) | 0.809  (0.535-1.221) | 0.312 |
| Albumin ≤ 4 / > 4 g/dL | 165/163 | 22.6 (15.2-30.0)/  55.6 (28.0-83.2) | 1.653  (1.253-2.181) | <0.001 |
| Bilirubin > 1.6 / ≤ 1.6 mg/dL | 20/312 | 29.5 (20.0-39.0)/  30.8 (22.6-39.0) | 1.012  (0.577-1.776) | 0.966 |
| ALT >40 / ≤ 40 U/L | 178/154 | 26.4 (21.1-31.7)/  42.7 (28.4-57.0) | 1.223  (0.929-1.611) | 0.152 |
| ALK-P >100 / ≤ 100 U/L | 132/199 | 21.0 (11.2-30.8)/  43.7 (26.8-60.6) | 1.527  (1.160-2.010) | 0.003 |
| GGT >60 / ≤ 60 U/L | 131/197 | 19.0 (11.2-26.9)/  46.5 (20.8-72.2) | 1.886  (1.434-2.480) | <0.001 |
| Platelet ≤ 10^5^ / > 10^5^ /mm^3^ | 51/262 | 30.1 (20.5-39.7)/  37.1 (24.2-50.0) | 1.055  (0.722-1.543) | 0.781 |
| ICG-15R > 10% / ≤ 10% | 160/170 | 28.7 (21.6-35.8)/  38.8 (16.0-61.6) | 1.345  (1.022-1.771) | 0.034 |
| HBeAg (Y/N) | 34/272 | 23.7(1.0-46.4)/  32.2(24.3-40.1) | 0.724  (0.482-1.088) | 0.120 |
| HBV genotype C/B | 138/174 | 27.9 (20.1-35.7)/  30.1 (18.2-42.0) | 1.181  (0.894-1.559) | 0.241 |
| HBV DNA >10^6^ / ≤10^6^ copies/mL | 136/184 | 24.2 (17.4-31.0)/  39.9 (26.4-53.4) | 1.320  (1.001-1.742) | 0.050 |
| HBsAg > 1000 / ≤1000 IU/mL | 121/157 | 24.2 (8.7-39.7)/  39.1 (26.1-52.1) | 1.207  (0.891-1.635) | 0.225 |
| G1896A mutation (Y/N) | 193/102 | 28.9 (18.2-39.6)/  27.9 (11.8-44.0) | 0.873  (0.650-1.172) | 0.367 |
| A1762T/G1764A mutation (Y/N) | 209/85 | 24.2 (16.6-31.8)/  29.5 (20.1-38.9) | 1.095  (0.790-1.517) | 0.585 |
| Anti-viral therapy (N/Y) | 292/40 | 26.6 (19.6-33.6)/  228.1 (NA) | 2.271  (1.494-3.452) | <0.001 |
| Tumor size > 5cm / ≤ 5cm | 122/210 | 10.9 (7.2-14.7)/  44.2 (31.1-57.3) | 1.858  (1.409-2.450) | <0.001 |
| Multi-nodularity (Y/N) | 141/191 | 16.0 (9.1-22.9)/  56.0 (24.5-87.5) | 1.995  (1.517-2.623) | <0.001 |
| Macroscopic venous invasion (Y/N) | 61/271 | 5.7 (3.4-8.0)/  39.1 (27.6-50.6) | 2.681  (1.923-3.745) | <0.001 |
| Cut margin ≤ 1cm/ >1cm | 223/108 | 27.9 (21.1-34.7)/  56.0 (24.9-87.1) | 1.399  (1.036-1.887) | 0.028 |
| AFP >20 / ≤ 20 ng/ml | 192/136 | 27.8 (15.8-39.8)/  38.8 (28.4-49.2) | 1.184  (0.894-1.568) | 0.239 |
| Microscopic venous invasion (Y/N) | 221/110 | 21.2 (13.0-29.4)/  61.4 (23.0-99.8) | 1.792  (1.321-2.427) | <0.001 |
| Cirrhosis (Y/N) | 143/179 | 28.7 (19.4-38.0)/  39.0 (9.4-68.6) | 1.394  (1.055-1.842) | 0.019 |
| Edmonson stage III or IV/ I or II | 214/108 | 19.7 (9.3-30.1)/  38.8 (29.8-47.8) | 1.349  (1.009-1.803) | 0.043 |
| BCLC stage B or C/ A | 147/182 | 12.6(6.2-19.0)/  46.5(31.8-61.2) | 1.825  (1.387-2.403) | <0.001 |

Abbreviations: ALT, alanine aminotransferase; AST, aspartate aminotransferase; Alk-P, alkaline phosphatase; GGT, gamma-glutamyltransferase; ICG-15R, indocyanine green retention rate at 15 minutes; HBsAg, hepatitis B surface antigen; BCP, basal core promoter; BCLC, the Barcelona-Clinic Liver Cancer; N: no; Y: yes
