# Supplementary material for: The Influence of Hepatitis B Viral Load and Pre-S Deletion Mutations on Post-Operative Recurrence of Hepatocellular Carcinoma and the Tertiary Preventive Effects by Anti-Viral Therapy
Source: PLoS One. 2013 Jun 21;8(6):e66457. doi: 10.1371/journal.pone.0066457 (PMC3689837; doi:10.1371/journal.pone.0066457)
Supplement: Table S5 — Multivariate analysis of factors associated with overall survival after resection for HCC. (DOCX) [file pone.0066457.s006.docx]

**Table S5. Multivariate analysis of factors associated with overall survival after resection for HCC**

| **Variable** | **Hazard ratio**  **(95% confidence interval)** | **Standard error** | | ***p*** |
| --- | --- | --- | --- | --- |
| **All patients (n=333)** | | | | |
| ICG-15R > 10% | 1.574 (1.122-2.208) | 0.173 | 0.009 | |
| Alk-P >100 U/L | 1.762 (1.267-2.450) | 0.168 | 0.001 | |
| Macroscopic venous invasion | 2.326 (1.580-3.425) | 0.197 | <0.001 | |
| Microscopic venous invasion | 1.848 (1.225-2.786) | 0.209 | 0.003 | |
| Without anti-viral therapy | 8.275 (2.631-26.023) | 0.585 | <0.001 | |
| **Patients without antiviral therapy after resection (n=271)** | | | | |
| ICG-15 > 10% | 1.618 (1.142-2.292) | 0.178 | | 0.007 |
| ALK-P >100 U/L | 1.441 (1.035-2.006) | 0.169 | | 0.031 |
| Tumor size > 5 cm | 1.602 (1.127-2.276) | 0.179 | | 0.009 |
| Macroscopic venous invasion | 2.155 (1.462-3.174) | 0.198 | | <0.001 |
| Microscopic venous invasion | 1.842 (1.209-2.801) | 0.214 | | 0.004 |
| Cirrhosis on non-tumor part | 1.486 (1.043-2.116) | 0.180 | | 0.028 |
| **Patients with available complete HBs gene sequence data but without anti-viral therapy (n=216)** | | | | |
| Age >60 years | 2.035 (1.288-3.215) | 0.233 | | 0.002 |
| Alk-P >100 U/L | 2.288 (1.451-3.606) | 0.232 | | <0.001 |
| Macroscopic venous invasion | 2.967 (1.730-5.102) | 0.276 | | <0.001 |
| Microscopic venous invasion | 1.825 (1.046-3.185) | 0.284 | | 0.034 |
| Pre-core (G1896A) mutation | 0.598 (0.382-0.937) | 0.229 | | 0.025 |

Abbreviations: ICG-15R, indocyanine green retention rate at 15 minutes; Alk-P, alkaline phosphatase
