# Supplementary material for: The Influence of Hepatitis B Viral Load and Pre-S Deletion Mutations on Post-Operative Recurrence of Hepatocellular Carcinoma and the Tertiary Preventive Effects by Anti-Viral Therapy
Source: PLoS One. 2013 Jun 21;8(6):e66457. doi: 10.1371/journal.pone.0066457 (PMC3689837; doi:10.1371/journal.pone.0066457)
Supplement: Table S6 — Univariate analysis of factors associated with overall survival after resection for hepatocellular carcinoma with available complete HBs gene sequence data. (DOCX) [file pone.0066457.s007.docx]

**Table S6.** **Univariate analysis of factors associated with overall survival after resection for hepatocellular carcinoma with available complete HBs gene sequence data**

| **Variable** | | **Number** | **Median survival months (95% CI)** | **Hazard ratio (95% CI)** | ***p*** |
| --- | --- | --- | --- | --- | --- |
| Age > 60 / ≤ 60 y/o | | 88/128 | 54.2 (43.2-65.2)/  125.9 (NA) | 1.821  (1.215-2.728) | 0.004 |
| Sex Female/Male | | 28/188 | 128.3 (27.3-229.3)/  81.9 (54.9-108.9) | 0.935  (0.510-1.714) | 0.829 |
| Albumin ≤ 4 / > 4 g/dL | | 106/106 | 50.7 (41.3-60.1)/  128.6 (NA) | 2.024  (1.329-3.083) | 0.001 |
| Bilirubin > 1.6 / ≤ 1.6 mg/dL | | 14/201 | 79.9 (48.3-111.5)/  100.0 (58.6-141.4) | 1.379  (0.602-3.155) | 0.448 |
| ALT >40 / ≤ 40 U/L | | 127/88 | 79.9 (50.8-109.0)/  100.9 (44.2-157.6) | 0.976  (0.645-1.477) | 0.909 |
| Alk-P >100 / ≤ 100 U/L | | 73/141 | 39.4 (25.4-53.4)/  100.9 (76.5-125.3) | 2.257  (1.504-3.387) | <0.001 |
| GGT >60 / ≤ 60 U/L | | 77/135 | 50.1 (36.3-63.9)/  100.9 (75.4-126.4) | 1.757  (1.171-2.638) | 0.006 |
| Platelet ≤ 10^5^ / > 10^5^ /mm^3^ | | 36/166 | 72.8 (36.0-109.6)/  94.5 (47.9-141.1) | 1.119  (0.681-1.840) | 0.656 |
| ICG-15R > 10% / ≤ 10% | | 111/103 | 55.5 (33.5-77.5)/  125.9 (NA) | 1.843  (1.209-2.809) | 0.004 |
| HBeAg (Y/N) | | 25/172 | 62.4(32.1-92.7)/  94.5(69.4-119.6) | 0.395  (0.429-1.396) | 0.395 |
| HBV genotype C/B | | 84/131 | 79.9 (50.5-109.3)/  125.9 (60.0-191.8) | 1.016  (0.676-1.526) | 0.941 |
| HBV DNA >10^6^ / ≤10^6^ copies/mL | | 103/112 | 72.8 (23.2-122.4)/  94.5 (62.7-126.3) | 1.265  (0.830-1.927) | 0.273 |
| HBsAg > 1000 / ≤1000 IU/mL | | 102/109 | 70.4 (45.1-95.7)/  100.0 (59.7-140.4) | 1.189  (0.786-1.799) | 0.412 |
| G1896A mutation (Y/N) | | 128/70 | 122.5 (81.3-163.7)/  53.7 (32.3-75.1) | 0.591  (0.388-0.900) | 0.013 |
| A1762T/G1764A mutation (Y/N) | | 135/63 | 72.8 (47.5-98.1)/  128.6 (NA) | 1.060  (0.665-1.688) | 0.807 |
| Pre-S deletion (Y/N) | | 73/143 | 54.5 (22.8-86.2)/  100.9 (56.2-145.6) | 1.384  (0.918-2.087) | 0.119 |
| Tumor size > 5cm / ≤ 5cm | | 66/150 | 66.7 (13.4-120.0)/  100.0 (50.4-149.6) | 1.639  (1.086-2.473) | 0.017 |
| Multi-nodularity (Y/N) | | 82/134 | 50.1 (37.0-63.2)/  125.9 (96.4-155.4) | 2.235  (1.488-3.355) | <0.001 |
| Macroscopic venous invasion (Y/N) | | 33/182 | 19.9 (0-46.0)/  113.5 (76.1-150.9) | 3.891  (2.410-6.289) | <0.001 |
| Cut margin ≤ 1cm/ >1cm | 146/69 | 71.2 (51.7-90.7)/  122.5 (92.8-152.2) | 1.473  (0.935-2.320) | 0.093 |  |
| AFP >20 / ≤ 20 ng/ml | | 117/95 | 72.8 (33.5-112.1)/  113.5 (55.3-171.7) | 1.464  (0.963-2.226) | 0.072 |
| Microscopic venous invasion (Y/N) | | 140/75 | 62.4 (43.0-81.8)/  128.3 (110.4-146.2) | 2.075  (1.284-3.356) | 0.002 |
| Cirrhosis (Y/N) | | 91/116 | 55.5 (38.1-72.9)/  125.9 (NA) | 1.781  (1.174-2.700) | 0.006 |
| Edmonson stage III or IV/ I or II | | 65/144 | 64.0 (36.6-91.4)/  94.5 (60.1-128.9) | 1.533  (1.009-2.330) | 0.044 |
| BCLC stage B or C/ A | | 84/129 | 54.2 (30.6-77.8)/  125.9 (68.1-183.7) | 1.924  (1.284-2.883) | 0.002 |

Abbreviations: ALT, alanine aminotransferase; AST, aspartate aminotransferase; Alk-P, alkaline phosphatase; GGT, gamma-glutamyltransferase; ICG-15R, indocyanine green retention rate at 15 minutes; HBsAg, hepatitis B surface antigen; BCP, basal core promoter; BCLC, the Barcelona-Clinic Liver Cancer; NA, not applicable; N: no; Y: yes
