# Supplementary material for: The Influence of Hepatitis B Viral Load and Pre-S Deletion Mutations on Post-Operative Recurrence of Hepatocellular Carcinoma and the Tertiary Preventive Effects by Anti-Viral Therapy
Source: PLoS One. 2013 Jun 21;8(6):e66457. doi: 10.1371/journal.pone.0066457 (PMC3689837; doi:10.1371/journal.pone.0066457)
Supplement: Table S7 — Univariate analysis of factors associated with recurrence after resection for hepatocellular carcinoma with available complete HBs gene sequence data. (DOCX) [file pone.0066457.s008.docx]

**Table S7. Univariate analysis of factors associated with recurrence after resection for hepatocellular carcinoma with available complete HBs gene sequence data**

| **Variable** | | **Number** | **Median recurrence months (95% CI)** | **Hazard ratio (95% CI)** | ***p*** |
| --- | --- | --- | --- | --- | --- |
| Age > 60 / ≤ 60 y/o | | 88/128 | 24.7 (14.5-34.9)/  37.1 (14.6-59.6) | 1.397  (0.991-1.970) | 0.055 |
| Sex Female/Male | | 28/188 | 32.2 (0.3-64.1)/  30.4 (20.6-40.2) | 0.883  (0.524-1.490) | 0.641 |
| Albumin ≤ 4 / > 4 g/dL | | 106/106 | 23.4 (16.7-30.1)/  52.1 (20.6-83.6) | 1.684  (1.190-2.385) | 0.003 |
| Bilirubin > 1.6 / ≤ 1.6 mg/dL | | 14/201 | 29.5 (23.6-35.4)/  31.0 (21.1-40.9) | 0.912  (0.463-1.797) | 0.790 |
| ALT >40 / ≤ 40 U/L | | 127/88 | 29.1 (17.1-41.1)/  32.2 (18.5-45.9) | 1.100  (0.775-1.562) | 0.593 |
| Alk-P >100 / ≤ 100 U/L | | 73/141 | 15.6 (4.3-26.9)/  39.9 (22.5-57.3) | 1.589  (1.119-2.256) | 0.009 |
| GGT >60 / ≤ 60 U/L | | 77/135 | 21.4 (9.0-33.8)/  39.1 (18.1-60.1) | 1.724  (1.221-2.434) | 0.002 |
| Platelet ≤ 10^5^ / > 10^5^ /mm^3^ | | 36/166 | 30.1 (17.6-42.6)/  38.8 (26.4-51.3) | 1.086  (0.684-1.721) | 0.727 |
| ICG-15R > 10% / ≤ 10% | | 111/103 | 29.5 (19.3-39.7)/  38.3 (12.6-64.0) | 1.360  (0.962-1.923) | 0.080 |
| HBeAg (Y/N) | | 25/172 | 25.4(1.5-49.3)  37.1(26.8-47.4) | 0.745  (0.451-1,231) | 0.248 |
| HBV genotype C/B | | 84/131 | 29.5 (18.9-40.1)/  31.0 (16.0-46.0) | 1.154  (0.817-1.629) | 0.415 |
| HBV DNA >10^6^ / ≤10^6^ copies/mL | | 103/112 | 25.4 (18.5-32.3)/  39.1 (26.9-51.5) | 1.637  (1.123-2.388) | 0.011 |
| HBsAg > 1000 / ≤1000 IU/mL | | 102/109 | 24.7 (13.2-36.2)/  34.6 (22.6-46.6) | 1.342  (0.913-1.972) | 0.135 |
| G1896A mutation (Y/N) | | 128/70 | 30.1 (18.5-41.7)/  27.9 (9.6-46.2) | 0.818  (0.567-1.181) | 0.282 |
| A1762T/G1764A mutation (Y/N) | | 135/63 | 29.5 (19.0-40.0)/  44.2 (0-110.5) | 1.294  (0.863-1.940) | 0.211 |
| Pre-S deletion (Y/N) | | 73/143 | 23.4 (14.2-32.6)/  38.7 (26.8-50.6) | 1.407  (0.991-1.996) | 0.054 |
| Tumor size > 5cm / ≤ 5cm | | 66/150 | 10.5 (4.8-16.2)/  38.8 (28.9-48.7) | 1.647  (1.153-2.353) | 0.006 |
| Multi-nodularity (Y/N) | | 82/134 | 14.1 (10.0-18.2)/  46.2 (15.5-76.9) | 2.033  (1.442-2.867) | <0.001 |
| Macroscopic venous invasion (Y/N) | | 33/182 | 5.5 (3.8-7.2)/  38.8 (28.9-48.7) | 3.226  (2.070-5.025) | <0.001 |
| Cut margin ≤ 1cm/ >1cm | 146/69 | 29.1 (21.0-37.2)/  56.0 (14.1-97.9) | 1.340  (0.918-1.957) | 0.127 |  |
| AFP >20 / ≤ 20 ng/ml | | 117/95 | 38.7 (26.2-51.2)/  26.9 (12.9-40.9) | 1.185  (0.836-1.680) | 0.338 |
| Microscopic venous invasion (Y/N) | | 140/75 | 21.4 (8.3-34.5)/  46.2 (14.4-78.0) | 1.639  (1.126-2.387) | 0.009 |
| Cirrhosis (Y/N) | | 91/116 | 27.9 (20.1-35.7)/  39.9(0-86.7) | 1.492  (1.047-2.125) | 0.026 |
| Edmonson stage III or IV/ I or II | | 65/144 | 19.7 (7.1-32.3)/  38.8 (29.0-48.6) | 1.410  (0.973-2043) | 0.068 |
| BCLC stage B or C/ A | | 84/129 | 12.6 (4.3-20.9)/  39.1 (28.1-50.1) | 1.648 (1.165-2.330) | 0.005 |

Abbreviations: ALT, alanine aminotransferase; AST, aspartate aminotransferase; Alk-P, alkaline phosphatase; GGT, gamma-glutamyltransferase; ICG-15R, indocyanine green retention rate at 15 minutes; HBsAg, hepatitis B surface antigen; BCP, basal core promoter; BCLC, the Barcelona-Clinic Liver Cancer; NA, not applicable; N: no; Y: yes.
